# Glutathione peroxidase 3 is a novel clinical diagnostic biomarker and potential therapeutic target for neutrophils in rheumatoid arthritis

**DOI:** 10.1186/s13075-023-03043-5

**Published:** 2023-04-22

**Authors:** Tao Chen, Zhen Zhou, Minge Peng, Huifang Hu, Rui Sun, Jiayi Xu, Chenxi Zhu, Yanhong Li, Qiuping Zhang, Yubin Luo, Bin Yang, Lunzhi Dai, Yi Liu, Luis E. Muñoz, Liesu Meng, Martin Herrmann, Yi Zhao

**Affiliations:** 1 Department of Rheumatology and Immunology, West China Hospital, Sichuan University, Chengdu, 610041 Sichuan China; 2 Clinical Institute of Inflammation and Immunology, Frontiers Science Center for Disease-Related Molecular Network, West China Hospital, Sichuan University, Chengdu, 610041 Sichuan China; 3 Chengdu Seventh People’s Hospital, Chengdu, 610041 Sichuan China; 4 Frontiers Science Center for Disease-Related Molecular Network, Sichuan University, Chengdu, 610041 Sichuan China; 5 Department of Laboratory Medicine, West China Hospital, Sichuan University, Chengdu, 610041 Sichuan China; 6 Department of Rheumatology and Immunology, National Clinical Research Center for Geriatrics and Department of General Practice, State Key Laboratory of Biotherapy, West China Hospital, Sichuan University, Chengdu, 610041 Sichuan China; 7 Department of Internal Medicine 3 - Rheumatology and Immunology, Friedrich-Alexander-University Erlangen-Nürnberg and Universitätsklinikum Erlangen, Erlangen, Germany; 8 Deutsches Zentrum Für Immuntherapie (DZI), Friedrich Alexander University Erlangen-Nuremberg and Universitätsklinikum Erlangen, Erlangen, Germany; 9 Institute of Molecular and Translational Medicine (IMTM), and Department of Biochemistry and Molecular Biology, Xi’an Jiaotong University Health Science Center, Shaanxi, Xi’an 710061 China

**Keywords:** Rheumatoid arthritis, Neutrophils, Hub genes, Reactive oxygen species

## Abstract

**Background:**

Neutrophils have a critical role in the pathogenesis of rheumatoid arthritis (RA) with immune system dysfunction. However, the molecular mechanisms of this process mediated by neutrophils still remain elusive. The purpose of the present study is to identify hub genes in neutrophils for diagnosis and treatment of RA utilizing publicly available datasets.

**Methods:**

Gene expression profiles were downloaded from the Gene Expression Omnibus, and batch-corrected and normalized expression data were obtained using the ComBat package. Gene Ontology and Kyoto Encyclopedia of Genes and Genomes enrichment analysis were used to conduct significantly functional analysis and crucial pathways. The resulting co-expression genes modules and hub genes were generated based on the weighted gene co-expression network analysis and visualization by Cytoscape. Flow cytometry was conducted to detect reactive oxygen species (ROS) levels in neutrophils.

**Results:**

Neutrophils underwent transcriptional changes in synovial fluid (SF) of RA patients, different from peripheral blood of healthy controls or patients with RA. Especially, glycolysis, HIF-1 signaling, NADH metabolism, and oxidative stress were affected. These hub genes were strongly linked with classical glycolysis-related genes (*ENO1*, *GAPDH*, and *PKM*) responsible for ROS production. The antioxidant enzyme glutathione peroxidase 3 (GPX3), a ROS scavenger, was first identified as a hub gene in RA neutrophils. Neutrophils from patients with autoinflammatory and autoimmune diseases had markedly enhanced ROS levels, most notably in RA SF.

**Conclusion:**

This research recognized hub genes and explored the characteristics of neutrophils in RA. Our findings suggest that the novel hub gene *GPX3* is involved in the neutrophil-driven oxidative stress-mediated pathogenesis of RA. It has the potency to be a target for neutrophil-directed RA therapy.

**Supplementary Information:**

The online version contains supplementary material available at 10.1186/s13075-023-03043-5.

## Introduction

Rheumatoid arthritis (RA) is a chronic autoimmune disorder with high titers of rheumatoid factor and anti-citrullinated protein antibodies (ACPAs) and elevated pro-inflammatory cytokines. These factors may lead to synovial hyperplasia, pannus formation, bone erosion, and eventually irreversible deformities [1, 2]. However, the complex pathogenesis of RA is incompletely understood. Currently, loss of immune tolerance, caused by the combined action of environmental and genetic factors, is regarded as a primary risk factor for developing RA [3, 4]. Immune tolerance is mainly brought on by phenotypic changes and abnormal activation of immune cells in innate and adaptive immunity. Among all immune cells, neutrophils have been recognized as critical players in different stages of RA progression, from leading to systemic immune tolerance loss to local synovial joint inflammation [5, 6]. Neutrophils contribute to RA in various ways: (I) they possess a pool of highly toxic proteases that are released at sites of inflammation (II) activated neutrophils can secrete a variety of pro-inflammatory cytokines and chemokines, causing subsequent inflammation (III) activated neutrophils form neutrophil extracellular traps, which contain externalized citrulline proteins as a source of modified autoantigens for the production of ACPAs [7].

Previous studies have reported that neutrophils from peripheral blood (PB) isolated from RA patients showed a functional difference from those of healthy donors. Compared with healthy circulating neutrophils, those from patients with RA displayed a higher production of reactive oxygen species (ROS), as well as a higher expression of Fc receptors [8, 9]. In RA synovial fluid (SF), it is accepted that abundant activated neutrophils with a specific phenotype were characterized by an increased oxidative burst, elevated cytokine secretion, and delayed apoptosis [10]. Increased hypoxia and glycolysis within RA joints are strongly associated with neutrophil activation and ROS production [11]. Synovial neutrophils also enhance the functions of resident fibroblast-like synoviocytes to secrete various inflammatory cytokines and to present antigen. Furthermore, they promote autoantibodies production [5]. Consequently, neutrophil activation is an important hallmark of RA, but the exact phenotype of PB neutrophils and SF neutrophils in RA is still elusive.

In the present study, we used a comprehensive integrative approach to analyze gene expression profiles and explored the characterization of neutrophils in PB and SF of patients with RA. We identified the differentially expressed genes (DEGs) of RA neutrophils based on the combined normalized data obtained for the GSE93776 [12], GSE116899 (unpublished data), and GSE154474 [10] gene expression data. Further studies to enrichment analysis of identified DEGs were performed to recognize biological functions and signaling pathways. Among these DEGs, the co-expression gene network was constructed to select the hub genes by weighted gene co-expression network analysis (WGCNA). In addition, we also collected neutrophils gene datasets with other autoinflammatory and autoimmune diseases, including systemic lupus erythematosus (SLE, GSE153781, unpublished data), adult-onset Still’s disease (AOSD, GSE150996) [13], and systemic juvenile idiopathic arthritis (SJIA, GSE122552) [14] to compare the oxidative burst of neutrophils. This work provides a broad insight into the neutrophils’ molecular features in developing RA on the transcriptome level. Together, these results support the view that neutrophils are important targets for the diagnosis and therapy of RA.

## Materials and methods

### Patients and samples

The study protocol was approved by the West China Hospital Ethics Committee of Sichuan University. The diagnosis of RA met the American College of Rheumatology (ACR) 2010 diagnostic criteria and the diagnosis of SLE fulfilled the 2019 ACR classification criteria. The diagnosis of adult-onset Still’s disease (AOSD) was based on the Yamaguchi criteria, after exclusion of infectious, hematologic, and autoimmune diseases. All patients signed the informed consent form and clinical characteristics of the patients were provided in Supplementary Table 1. Human blood samples from RA patients, SLE patients, and normal healthy donors and SF samples from RA patients were enrolled from in-patient and physical examination volunteers of West China Hospital, Sichuan University, from May to August 2022. All samples were stored at 4 °C until neutrophil isolation on the same day.

### Neutrophil isolation and ROS detection

Neutrophils from SF were harvested by centrifugation at 4000 rpm for 10 min at 4 °C [15]. The cells were then filtered on a 40-μm cell strainer and centrifuged at 300 g 4 °C for 5 min. Blood neutrophils were collected by Ficoll-Hypaque 1.077 density gradient sedimentation [16, 17]. The cell layer at the junction of the third and fourth layers was collected. Erythrocytes were eliminated by hypotonic water lysis with a 20-s exposure to ice-cold distilled water quickly added with 0.1 × volume of 10 × PBS and 5 min centrifugation at 300 g 4 °C [18]. Finally, cell pellets were washed with 1xPBS once and re-suspended in 1xPBS for cell counting. Isolated neutrophils (5 × 10^6^/ml) were treated with 20 μM dichlorofluorescin diacetate (DCF-DA, Sigma) for 30 min at 37 °C in RPMI-1640 media containing 10% FBS. Intracellular ROS level was detected by measuring the mean fluorescence intensity (MFI) of neutrophils gated on FSC/SSC on a flow cytometry (FCM).

### Quantitative real time PCR (qPCR)

Total RNA was extracted from neutrophils using the RNeasy® Mini Kit (Qiagen, Crauley, UK) according to the manufacturer’s protocol. One microgram of RNA was used to generate cDNA by a HiScript® III 1st Strand cDNA Synthesis Kit (Vazyme, China). Transcripts were quantified by real-time qPCR using ChamQ SYBR qPCR Master Mix (Vazyme, China) on a CFX96 Real-time system (Bio-Rad, USA). The relative gene-expression levels were calculated with ΔΔCt method using β-actin gene expression as reference. The primer sequences used were as follows: GPX3, 5′-TACGGAGCCCTCACCATTGATG-3’ ′(forward) and 5′-CAGACCGAATGGTGCAAGCTCT-3′ (reverse); ENO1, 5′-AGTCAACCAGATTGGCTCCGTG-3′ (forward) and 5′-CACAACCAGGTCAGCGATGAAG-3′ (reverse); ALDOA, 5′- GACACTCTACCAGAAGGCGGAT-3′ (forward) and 5′- GGTGGTAGTCTCGCCATTTGTC-3′ (reverse); PKM, 5′-ATGGCTGACACATTCCTGGAGC-3′ (forward) and 5′- CCTTCAACGTCTCCACTGATCG-3′ (reverse); GAPDH, 5′- GTCTCCTCTGACTTCAACAGCG-3′ (forward) and 5′-ACCACCCTGTTGCTGTAGCCAA-3′ (reverse); CYBB, 5′- CTCTGAACTTGGAGACAGGCAAA-3′ (forward) and 5′- CACAGCGTGATGACAACTCCAG-3′ (reverse); β-actin, 5′- CACCATTGGCAATGAGCGGTTC-3′ (forward) and 5′- AGGTCTTTGCGGATGTCCACGT-3′ (reverse).

### Microarray data

All data used in this study were obtained from publicly available data. To characterize neutrophils in RA patients with PB and SF, microarray datasets (GSE116899, GSE93776 and GSE154474) were downloaded from Gene Expression Comprehensive Database (GEO; http://www.ncbi.nlm.nih.gov/geo/) [19]. To determine the DEGs in neutrophils associated with autoinflammatory and autoimmune diseases, these expression profiles also were obtained from GEO (GSE153781, GSE122552, and GSE150996). These microarray data were extracted and evaluated, according to the platforms and number of samples, including PB neutrophils in both RA, SLE, SJIA, AOSD and healthy controls, and SF neutrophils in RA. Details of these sequencing databases are shown in Table 1.

**Table 1:** Details for GEO RA data

Author	GEO accession	Methods	Platform	Sample (neutrophil)					
				HC PB	RA PB	RA SF	SLE PB	AOSD PB	SJIA PB
Goldberg G	GSE116899	RT-PCR microarrays	GPL11154	5	7	3	/	/	/
Tasaki S	GSE93776	RT-PCR microarrays	GPL570	6	9	/	/	/	/
Wright HL	GSE154474	RT-PCR microarrays	GPL11154	/	*3*	*3*	*/*	*/*	*/*
Xuan Z	GSE153781	RT-PCR microarrays	GPL20795	*6*	*/*	*/*	*6*	*/*	*/*
Jia J	GSE150996	RT-PCR microarrays	GPL23227	*6*	*/*	*/*	*/*	*6*	*/*
Schulert GS	GSE122552	RT-PCR microarrays	GPL23934	*5*	*/*	*/*	*/*	*/*	*6*

### Differential expression analysis

GEO2R (http://www.ncbi.nlm.nih.gov/geo/geo2r) analysis was used for detecting RA-associated neutrophils’ DEGs in PB and SF, compared with healthy control [20]. Significant DEGs were identified with adjusted *P-value* < 0.05 and |logFC|> 1 (logFC > 1 for upregulated genes; logFC <  − 1 for downregulated genes). The online website (http://vip.sangerbox.com/login.html) was used to plot principal component analysis (PCA), volcano plots, heat maps,and Venn diagrams [21, 22].

### Functional enrichment analysis

Gene Ontology (GO) enrichment and the Kyoto Encyclopedia of Genes and Genomes (KEGG) pathways analysis were performed to identify significantly enriched gene sets with the packages org.Hs.eg.db (version 3.1.0) and clusterProfiler (version 3.14.3), respectively [23]. The maximum gene set size was set at 5000 genes and the minimum size at 5 genes. *P-value* of < 0.05 were considered statistically significant.

### Gene co-expression network construction

Co-expression gene modules correlated to the clinical traits were constructed and identified using the WGCNA R package [24]. Normalized data were analyzed to generate co-expression modules using the function blockwise Module for automatic network construction with default parameters in WGCNA. Then, a soft threshold value of *β* = 16 was used to derive the adjacency matrix and topological overlap matrix to minimize the effects of noise and spurious associations. Following that, genes with similar co-expression patterns across samples were clustered based on the topological overlap dissimilarity measure with a minimum gene module size of 30. Pearson’s correlation coefficient analysis was performed to select the interesting module, combined with the correlative clinical characteristics. For the interesting module, the hub genes were defined based on module membership (MM) > 0.8 and gene significance (GS) > 0.1. Finally, the gene co-expression network was visualized utilizing Cytoscape (version 3.8.2).

### Statistical analysis

Statistical analysis was performed using the GraphPad Software. Graphs were generated by GraphPad Prism 9 and FlowJo v10. Two groups of data were compared using Student’s *t*-test. *P*-value < 0.05 indicated statistically significant.

## Results

### Study characteristics

**Fig. 1:**
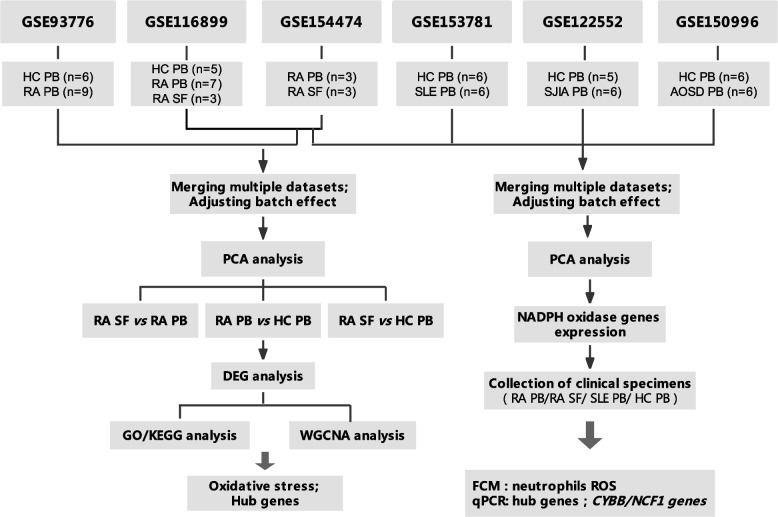
Study design and methodologies

In this study, we analyzed the heterogeneity of neutrophils in PB and SF of RA patients based on three datasets and elucidated from multiple aspects, including transcript level, GO/KEGG enrichment analysis, co-expression module genes construction, and hub genes identification. At the same time, it has also been analyzed and clinically verified with neutrophils of autoinflammatory and autoimmune diseases, including RA, SLE, AOSD, and SJIA. The study flow chart is shown in Fig. 1.

### The transcriptional signature of neutrophil in RA

**Fig. 2:**
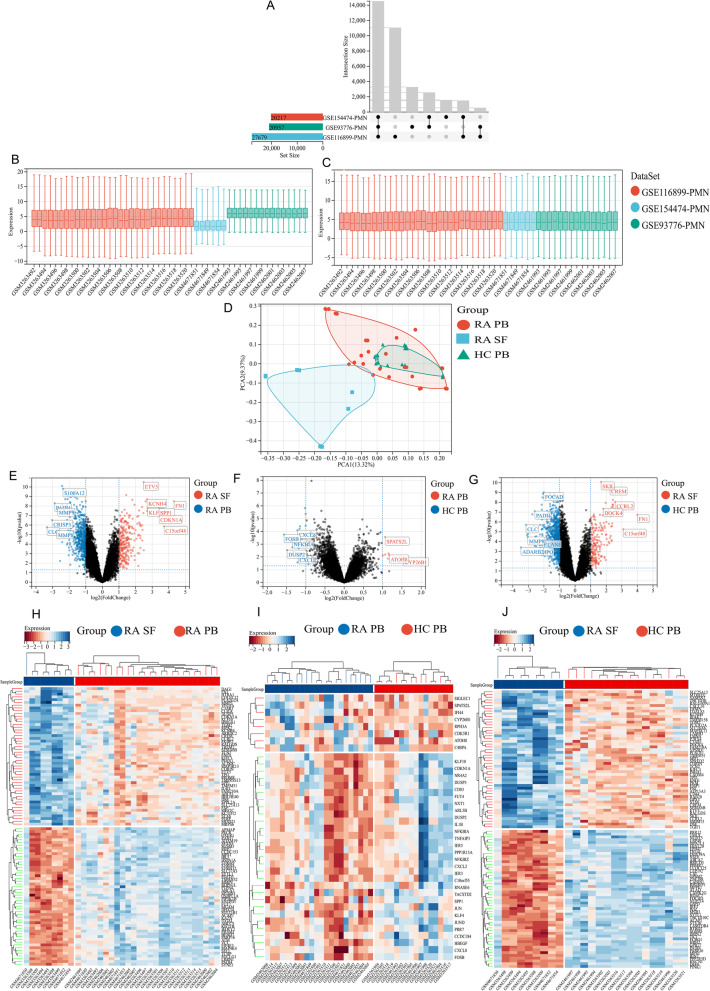
Transcriptomic analysis of neutrophils in RA patients compared with HC (control). **A** Venn diagram representing shared genes from three mRNA expression profiling datasets GSE154474, GSE93776, and GSE116899. **B**, **C** Box plots visualization of GSE154474, GSE93776, and GSE116899 data pre- and post-normalization. **D** PCA of normalized expression data. Volcano plots and heat maps of DEGs based on normalized data: **E**, **H** DEGs between RA SF and RA PB; **F**, **I** DEGs between RA PB and HC PB; **G**, **J** DEGs between RA SF and HC PB

To obtain the normalized matrix, we merged datasets (GSE116899, GSE93776, and GSE154474) and adjusted batch effects. The Venn diagram showed three datasets shared 14,536 genes (Fig. 2A). From the boxplots, we could observe that the sample distribution of these datasets was quite different (Fig. 2B). After adjusting the batch effects, the distribution of data across datasets tended to be consistent, suggesting that batch effects were removal (Fig. 2C).

First, PCA analysis was used to visualize normalized genes expression profiles by reducing the dimension. The cluster of RA SF neutrophils was significant; however, there was no distinct separation between RA PB and HC PB (Fig. 2D). Next, we performed differential gene analysis on this standard dataset. The fold change and *P-value* of DEGs were displayed in volcano plots (Fig. 2E–G). Heat maps showed that RA SF neutrophils had significant DEGs compared with RA PB or HC PB (Fig. 2H, J). However, there were no significant differences between RA PB and HC PB neutrophils (Fig. 2I). This indicated that the level of the transcriptome in SF neutrophils was highly elevated, participating in the pathogenesis of RA.

### The oxidative stress of neutrophil in RA

According to the screening criterion of |logFC|> 1 and *P*-value < 0.05, 781 DEGs were screened out, containing 265 upregulated and 516 downregulated genes through the comparison of PB and SF of the RA neutrophils. Compared to HC PB, 37 DEGs were identified in RA PB, and 873 DEGs were identified in RA SF with 194 upregulated genes and 679 downregulated genes. Next, we conducted GO terminology and KEGG pathway enrichment analysis for these DEGs, respectively.

**Fig. 3:**
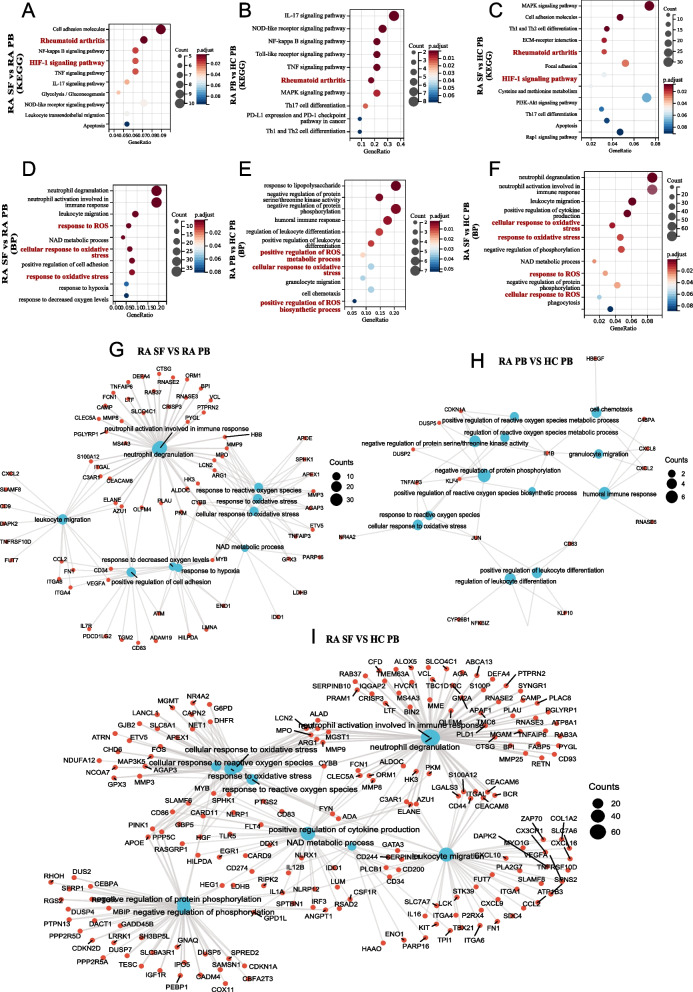
GO enrichment and KEGG pathway analyses. KEGG enrichment analysis of DEGs under group: **A** “RA SF vs. RA PB”; **B** “RA PB vs. HC PB”; **C** “RA SF vs. HC PB”; GO enrichment analysis of DEGs under group: **D** “RA SF vs. RA PB”; **E** “RA PB vs. HC PB”; **F** “RA SF vs. HC PB”; **G**–**I** network visualization of enriched pathways (GO/KEGG) in the gene signature

When we examined the enriched GO terms or KEGG pathways, there were common important GO terms and KEGG pathways were significantly enriched in the RA SF and RA PB, such as rheumatoid arthritis, neutrophils activation, response to reactive oxygen species, and response to oxidative stress (Fig. 3A–F). In RA (KEGG: 05,323), 13 DEGs genes were confirmed to be involved. Among them, the MHC class II HLA genes (HLA-DRA, HLA-DRB1, HLA-DMA, and HLA-DMB) were critical restriction elements for CD4^+^ T cells proliferation and differentiation; cytokines (IL1A, IL1B, CCL2, VEGFA, and MMP3) mediated inflammation and modulate immunity; and costimulatory molecule CD86 was essential for full activation of naive T cell and subsequent differentiation. Besides, there were still uniqueness. RA SF neutrophils were also involved in the HIF-1 signaling pathway, in which the property of neutrophils may be conferred by the local hypoxic environment in the RA joint (Fig. 3A, C). However, neutrophils in RA SF and RA PB were both involved in Th1, Th2, and Th17 cell differentiation and MAPK signaling, which mediated adaptive immunity response (Fig. 3B, C). Finally, we also performed a GO/KEGG visualization of neutrophil-mediated immunity and ROS-related entries (Fig. 3G–I). These results suggested the importance of neutrophils-mediated immune response and oxidative stress in RA neutrophils.

### Identification of the hub genes in RA neutrophils

**Fig. 4:**
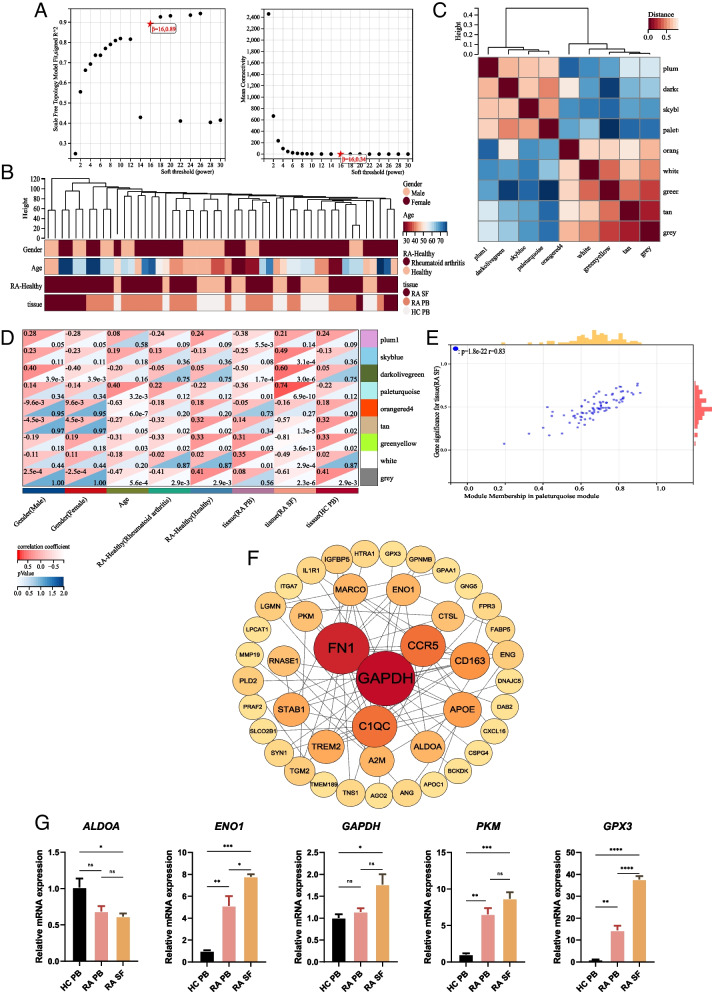
Co-expression network constructed by WGCNA based on the combined data from GSE154474, GSE93776, and GSE116899. **A** The soft-thresholding power (β) of 16 was chosen based on the scale-free topology criterion in normalized data from (**B**) clustering dendrogram of samples with clinical traits heatmap. **C** Identification of meta-module and relationships among module-module. **D** Heatmap showing module-trait relationship of magenta module and clinical traits (columns). **E** Scatter plot of gene significance (GS) for RA SF vs. module membership (MM) in the co-expression paleturquoise module. **F** Construction and visualization of a WGCNA co-expression network by Cytoscape software and screening hub genes. **G** *ALDOA*, *ENO1*, *GAPDH*, *PKM*, and *GPX3* mRNA levels were determined by qPCR

We performed WGCNA, to further clarify the co-expression network and hub genes of RA neutrophils. A soft threshold power of *β* was set to 16 to construct a scale-free network (Fig. 4A). This normalized gene expression data with clinical information were clustered into nine main modules represented by different color (dark olive green, green yellow, grey, orange red4, paleturquoise, plum1, sky blue, tan, white) (Fig. 4B, C). We calculated the correlation between modules and clinical characteristics, such as gender, age, health, RA, RA PB, RA SF, and HC PB, to investigate the clinical significance of modules (Fig. 4D). The paleturquoise module was highly positively correlated with RA SF (*p* = 1.8e–22, *r* = 0.83) (Fig. 4E). Therefore, we selected pale turquoise as key module for the consecutive study and used Cytoscape to construct a gene interaction network containing 50 genes of the paleturquoise module (Fig. 4F). Finally, we calculated the correlation between the module feature vector and gene expression to obtain MM. Based on the cut-off criteria (| MM|> 0.8), these genes in the paleturquoise module were identified as hub genes, including *ENO1*, *GAPDH*, *ALDOA*, *PKM*, and *GPX3*. Further, we validated the mRNA expression of hub genes in RA neutrophils by qPCR. The results showed that the expressions of *ENO1*, *PKM*, and *GPX3* in paired PB and SF of RA neutrophils were considerably higher than those of healthy group, and their expression was further increased in RA SF (Fig. 4G). This finding indicated that hub genes (*ENO1*, *PKM*, and *GPX3*) were positively inter-correlated with RA.

### Neutrophils from patients with autoinflammatory and autoimmune diseases showed an increased ROS production

**Fig. 5:**
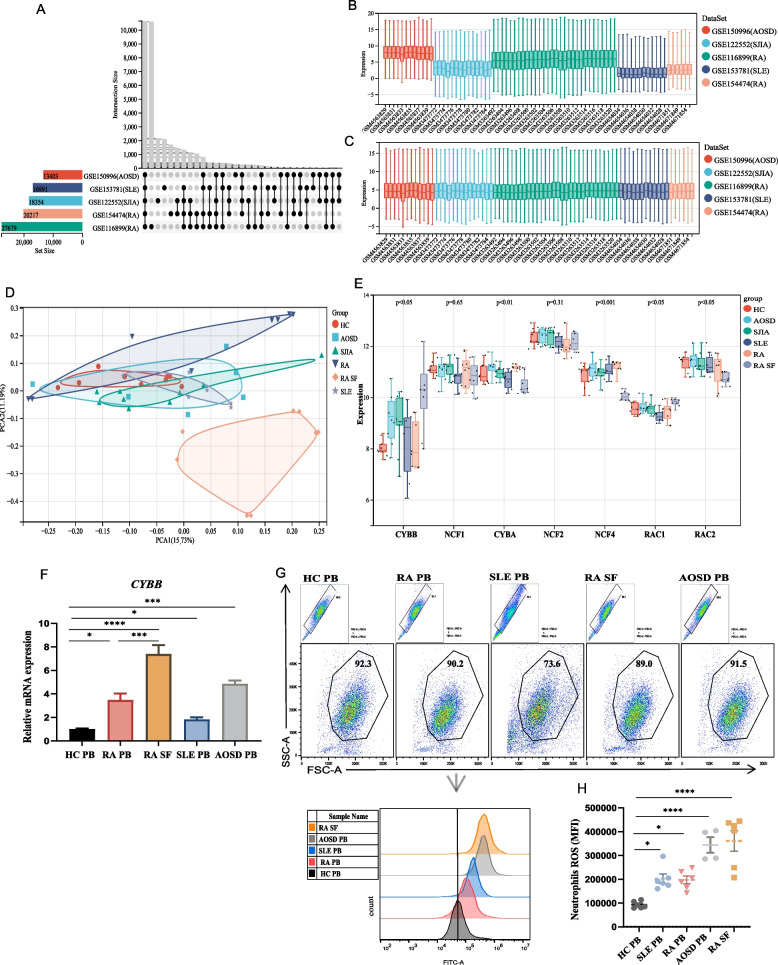
Transcriptomic analysis of neutrophils in autoimmune disease patients compared with HC (control). **A** Venn diagram displaying the overlap of the five gene expression datasets, including RA (GSE116899 and GSE15474), SLE (GSE153781), SJIA (GSE122552), and AOSD (GSE150996). **B**, **C** Box plots of expression data before and after normalization. **D** PCA plot analysis of normalized data. **E** The expression levels of NADPH oxidase-related genes in normalized data. **F** The mRNA expression level of *CYBB* was detected by qPCR. FCM analysis of ROS MFI in neutrophils with HC, SLE, and RA: **G** MFI was calculated using FlowJo software. **H** Statistical analysis was performed in Prism

We explored whether neutrophil-driven oxidative stress was also observed in other autoinflammatory and autoimmune diseases. Besides RA (GSE116899 and GSE154474), we also analyzed gene expression profiles for neutrophil-associated autoinflammatory and autoimmune diseases from public data, including SLE (GSE153781), SJIA (GSE122552), and AOSD (GSE150996). Upon merging these five datasets, we obtained 10,659 intersected genes (Fig. 5A). After adjusting for batch effects, we obtained normalized gene expression data (Fig. 5B, C). Following gene-expression normalization, PCA showed RA SF neutrophils clearly segregated from others, but other samples exhibited lower separation from each other. PCA indicated a higher heterogeneity in the RA SF neutrophils compared with other autoinflammatory and autoimmune diseases (Fig. 5D).

In neutrophils, ROS were mainly generated via assembly of the NADPH oxidase 2 (NOX2) complex, consisted of p47phox (NCF1), p67phox (NCF2), p40phox (NCF4), p22phox (CYBA), and gp91phox (CYBB). When neutrophils were activated, the cytosolic components (NCF1, NCF2, NCF4, RAC1, and RAC2) migrated to the membranes, where they associated with the membrane-bound components (CYBB and CYBA) to form an active enzyme [25]. Researching on mutations causing human chronic granulomatous disease showed that mutations in NCF1 and CYBB could result in a complete lack of function of the NOX2 complex [26]. Compared with HC PB, *CYBB* expression levels had a significant upregulated in SLE PB, SJIA PB, AOSD PB, and RA SF (Fig. 5E). Among them, RA SF neutrophils showed the highest expression level of *CYBB* (Fig. 5E). However, heat map showed expression levels of *NCF1*, *CYBA*, *NCF2*, *NCF4*, *RAC1*, and *RAC2* had no significant differences among these groups (Fig. 5E). In addition, we also verified the mRNA expression of *CYBB* in neutrophils with autoinflammatory and autoimmune diseases. The results showed that *CYBB* expression was increased in RA SLE, and AOSD, indicating CYBB may play an important role in autoinflammatory and autoimmune diseases through mediating oxidative stress of neutrophils.

Next, we quantified ROS levels from PB neutrophils of HC, SLE and RA, and SF from RA patients by FCM assay with DCFH-DA staining. The results of FCM analysis revealed a significantly higher ROS content in the neutrophils from the disease groups when compared to the healthy group (Fig. 5G, H). Interestingly, neutrophils from SF of patients with RA exhibited higher ROS levels than neutrophils from matched PB. Our results revealed that an increased oxidative burst of neutrophils was closely related to autoinflammatory and autoimmune diseases. The underlying molecular mechanisms still need further exploration.

## Discussion

Neutrophils are recognized as key immune cells in the pathogenesis and progression of RA. They orchestrate systemic inflammation that breaks immune tolerance and causes joint destruction. A better systematic and comprehensive understanding of the heterogeneity of neutrophils in health and disease constitutes an avenue to identify further potential biomarkers associated with RA.

In this work, we performed DEGs analysis of neutrophils isolated from PB and SF of patients with RA and PB of HC from the normalized data (GSE116899, GSE154474, and GSE93776). Compared with HC PB neutrophils, 873 DEGs and 37 DEGs were identified in SF and PB with RA, respectively. In patients with RA, 781 DEGs in SF neutrophils were obtained compared with PB neutrophils. PCA, volcano maps, and heat maps all showed that substantial gene expression differences in neutrophils from SF of RA patients. The differences in neutrophils of PB from patients and controls were less pronounced. In other words, the gene expression of neutrophils in the SF is strongly affected.

Next, we performed GO and KEGG pathway analysis on the normalized data. A large number of inflammatory signaling pathways are acting in RA SF neutrophils. These comprise the following signaling pathway: (I) NF-kappa B, (II) HIF-1, (III) TNF, (IV) IL-17, (V) RA, (VI) NOD-like receptor (VII) neutrophils activation. Also, the metabolic and biosynthetic pathways were affected like (I) glycolysis/gluconeogenesis, (II) NAD metabolic process, (III) response to oxidative stress, and to (IV) ROS when compared with the matched neutrophils of the peripheral blood. Differentiation to the T cell subsets Th1, Th2, and Th17 appeared more advanced in RA SF and PB than in HC PB neutrophils. These results illustrated that in the RA SF activated neutrophils are altered most likely due to the unique microenvironment in the synovium. These neutrophils played a pivotal role in the initial events leading to the pathogenesis of RA.

Importantly, there is an urgent demand for identifying potential neutrophil-associated biomarkers or therapeutic targets for RA. We used the WGCNA method to identify nine co-expression modules with clinical features (gender, age, RA, healthy, tissue), based on the standardized data. Among these, the paleturquoise module was positively associated with SF in RA. It consisted of 84 genes and ten of these were identified as hub genes. The hub genes *ENO1*, *ALDOA*, *GAPDH*, and *PKM* were involved in NADH metabolic process and glycolysis/gluconeogenesis. ENO1, a critical glycolytic enzyme, has been reported to be highly expressed on monocytes, macrophages, and fibroblasts-like synoviocytes from patients with RA. It causes the production of synovial inflammatory factors [27, 28]. The glycolytic enzymes GAPDH regulated the glucose oxidation through glycolysis or the pentose phosphate pathway (PPP). The latter results in accumulation of NADPH and NOX2-dependent ROS generation [29]. TNF-α stimulates the upregulation of the glycolytic gene GAPDH was seen in RA-FLS compared to healthy controls [30]. Another key glycolytic enzyme, PKM, was involved in the progression of RA. It triggered the activation of macrophages and the production of pro-inflammatory cytokines [31]. Here, we hypothesized that under low tissue oxygen tensions in the RA SF, neutrophils initiate oxidative stress, activate HIF-1 signaling, and reprogram the glucose metabolism pathway towards PPP to generate more ROS. This has also been observed in neutrophils infiltrating hypoxic myocardium after acute myocardial infarction [32]. The systematic screening of glycolysis-related genes in synovial neutrophils of patients with RA provided the theoretical basis for future investigations with specific pathway inhibitors.

The imbalance between ROS generation and the antioxidant system triggered oxidative stress in patients with cancer, autoimmune rheumatic diseases, and further diseases [33–35]. In order to maintain the redox homeostasis, some anti-oxidant genes are compensatorily upregulated and scavenge excessive ROS. Hub gene *GPX3*, a member of the glutathione peroxidase family of selenoproteins, reduces extracellular ROS level and, thus, protects host cells from oxidative damage [36]. GPX3 modulates the ROS level in malignant melanoma, hepatocellular carcinoma, and colitis-associated carcinoma and consequently inhibits tumor cell proliferation [37–39]. GPX3 serum levels were employed as biomarker for the recurrence of lung cancer after complete resection [40]. To sum up, we identified the hub gene *GPX3* as a novel predictive biomarker in diseases associated with oxidative stress, such as RA.

Finally, we also analyzed the ROS production of neutrophils associated with the autoinflammatory and autoimmune diseases RA, SLE, AOSD, and SJIA, from publicly available data. NADPH oxidase CYBB, required for ROS production, displayed an increased expression in SF neutrophils when compared to matched neutrophils from PB (RA, as well as in other immune diseases). To test this, we isolated neutrophils from clinical samples and examined their ROS levels. The results showed that the ROS levels in the disease group (SLE, AOSD, and RA) were higher than those in the HC, in which the ROS in SF neutrophils significantly increased. Although the ROS of neutrophils was enhanced, previous studies have reported that neutrophil-driven ROS employ different pathogenic mechanisms in RA and SLE. Increased hypoxia and glycolysis drove neutrophil activation and ROS-mediated NETs production in RA, while decreased redox capacity increased ROS-mediated damage in SLE [11]. In the microenvironment of RA SF, the neutrophil transcriptome was changed during migration into inflamed joints. Here, neutrophils participate in the inflammatory response and enhanced their ROS production. Clarifying this path helps in developing an approach to target neutrophils within the joint dependent on the degree of inflammation. The drawback of this study part was that we were not able collect sufficient samples from SJIA patients to verify the conclusion.

Taken together, we identified the upregulation of neutrophil-specific genes in RA that can serve as biomarkers for RA. Further investigations into other systemic inflammatory diseases might shed light on the specificity of these genes. The main limitation of this study was the small number of clinical samples in each group for validating hub genes. Therefore, in future studies, this limitation should be fully considered to further verify findings of this study.

## Conclusions

With bioinformatic network analyses, we found that neutrophil-specific hub genes were highly expressed in SF neutrophils of patients with RA. These specific central genes are highly correlated to several signaling pathways like (I) neutrophil activation, (II) inflammation, (III) HIF-1, (IV) glycolysis/gluconeogenesis, and (V) NADH metabolic process. A novel hub gene *GPX3* may be the potential biomarker for diagnosis and treatment of RA. These findings reinforce our understanding of the underlying molecular pathogenetic role of neutrophils in RA and provide insights for the development of neutrophil-targeted therapies.

## Supplementary Information

**Additional file 1: Table 1. **Clinical characteristics of patients for this study.

## Data Availability

The data that support the findings of this study are openly available and can be downed from GEO (https://www.ncbi.nlm.nih.gov/geo/).

## Abbreviations

RA: Rheumatoid arthritis
ROS: Reactive oxygen species
GPX3: Glutathione peroxidase 3
SF: Synovial fluid
ACPAs: Anti-citrullinated protein antibodies
PB: Peripheral blood
DEGs: Differentially expressed genes
WGCN: Weighted gene co-expression network analysis
SLE: Systemic lupus erythematosus
AOSD: Adult-onset Still’s disease
SJIA: Systemic juvenile idiopathic arthritis
PCA: Principal component analysis
GO: Gene ontology
KEGG: Kyoto encyclopedia of genes and genomes
MM: Module membership
GS: Gene significance
ACR: American college of rheumatology
DCF-DA: Dichlorofluorescin diacetate
MFI: Mean fluorescence intensity
FCM: Flow cytometry
qPCR: Quantitative real time PCR
HC: Healthy control
NOX2: NADPH oxidase 2
PPP: Pentose phosphate pathway
