# Supplementary material for: Glutathione peroxidase 3 is a novel clinical diagnostic biomarker and potential therapeutic target for neutrophils in rheumatoid arthritis
Source: Arthritis Res Ther. 2023 Apr 22;25:66. doi: 10.1186/s13075-023-03043-5 (PMC10122307; doi:10.1186/s13075-023-03043-5)
Supplement: Supplementary file 1 — Additional file 1: Table 1. Clinical characteristics of patients for this study. [file 13075_2023_3043_MOESM1_ESM.docx]

**Supplementary Table 1.** Clinical characteristics of patients included in the study.

| Patient ID | Age (Year) | Gender | Disease duration (Moth) | CRP (mg/L) | RF status | Anti-DNA status | Medications | DAS-28 | SLEDAI-2K |
| --- | --- | --- | --- | --- | --- | --- | --- | --- | --- |
| RA-1 | 52 | F | 12 | 48.2 | + | - | No | 3.01 | / |
| RA-2 | 53 | F | 72 | 49.8 | + | - | Leflunomide, Chloroquine | 5.49 | / |
| RA-3 | 30 | F | 144 | 20.4 | + | - | Methotrexate,Chloroquine | 3.88 | / |
| RA-4 | 66 | F | 120 | 73.6 | + | - | Tofacitinib，Methotrexate | 2.51 | / |
| RA-5 | 57 | F | 96 | 40.9 | + | - | Prednisone | 2.30 | / |
| RA-6 | 64 | F | 108 | 10.6 | + | - | No | 3.80 | / |
| SLE-1 | 44 | F | 240 | 21.3 | - | + | Prednisone，Tacrolimu，Mycophenolate mofetil | / | 4 |
| SLE-2 | 53 | F | 240 | 3.42 | - | + | Prednisone, Chloroquine | / | 16 |
| SLE-3 | 33 | F | 204 | 7.16 | - | - | Prednisone, Cyclophosphamide | / | 10 |
| SLE-4 | 27 | F | 108 | 1.42 | - | - | Methylprednisolone, Mycophenolate mofetil, Chloroquine, Belizumab | / | 10 |
| SLE-5 | 20 | F | 3 | 79 | - | - | Metacortandracin, Chloroquine, Tacrolimus | / | 8 |
| SLE-6 | 52 | F | 168 | 26.4 | - | + | Methylprednisolone, Mycophenolate mofetil | / | 8 |
| AOSD-1 | 57 | F | 2 | 218 | - | - | Dexamethasone, Ciclosporin A | / | / |
| AOSD-2 | 18 | M | 6 | 30.3 | - | - | Prednisone | / | / |
| HC1-6 | 25-65 | F | 0 | 0 | - | - | No | / | / |
